# License to Replicate: Mechanisms of Licensing Eukaryotic Origins for DNA Replication

**DOI:** 10.1002/bies.70095

**Published:** 2025-12-21

**Authors:** Victoria Frisbie, Franziska Bleichert

**Affiliations:** ^1^ Department of Molecular Biophysics and Biochemistry Yale University New Haven USA

## Abstract

Accurate and timely genome replication is a universal feature of living organisms and a prerequisite for cell proliferation. In eukaryotes, genomic DNA replication initiates in two steps, origin licensing and origin firing, reflecting the loading and activation of the MCM replicative helicase motor on origin DNA. Biochemical reconstitution of these steps in the budding yeast model system signified a major advance towards understanding the molecular and structural details of eukaryotic replication initiation events. With recent successes in reconstituting origin licensing with purified human proteins, a mechanistic picture is beginning to emerge on how DNA replication initiates in higher eukaryotes and how it deviates from the paradigms established in budding yeast. In this review, we highlight similarities and differences in licensing mechanisms between yeast and metazoa.

## Graphical abstract

Origin licensing constitutes the first step in initiating eukaryotic genome replication. Licensed origins are marked by an MCM double hexamer complex. This article reviews the molecular mechanisms of MCM double hexamer loading in yeast and higher eukaryotes, based on insights gained from biochemical reconstitution and structural analyses.

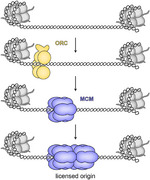

## 1 Challenges for Genome Replication

The replication of chromosomal DNA is essential to sustaining cell proliferation across the three domains of life. Maintaining stable genomes requires that replication events are complete and highly accurate, with the entire genome being duplicated exactly once with minimal mistakes to avoid gene and chromosome copy number changes and the accumulation of mutations [1, 2]. Replication starts at genomic sites known as replication origins and proceeds bidirectionally [3, 4, 5, 6]. While many prokaryotes replicate their chromosome from a single origin, larger genomes initiate replication at multiple sites to ensure all DNA is replicated in a timely manner [7, 8, 9]. For example, it would take ∼58 days to replicate human chromosome 1 (the largest chromosome in human cells with 249 million base pairs) from a single origin assuming a replication fork speed of 1.5 kb/min [10]. Thus, origin number scales with genome size, ranging from a few hundred origins in budding yeast to tens of thousands of origins in human cells [11, 12, 13, 14, 15, 16].

In eukaryotic cells, chromosome replication faces several additional challenges. First, replication onset from the many start sites must be coordinated and origins must be appropriately distributed across the genome so that no DNA is left unreplicated in a given cell cycle [2]. Genomic regions sparse of replication initiation events are associated with common fragile sites and are prone to breakage due to incomplete DNA replication [17, 18, 19]. Second, cells must limit genome replication to once per cell cycle, preventing re‐initiation of replication in already duplicated DNA regions while permitting these events at origins in non‐replicated regions [20]. Eukaryotic cells solve this problem by tightly integrating the onset of DNA replication with cell cycle cues [21]. Disruption of this regulation leads to re‐replication and genome instability, and is frequently seen in cancer cells [22, 23]. Third, DNA replication must cope with molecular crowding in the nucleus, other DNA‐templated transactions, and chromatin [24]. As for chromatin, nucleosomes present an obstacle to DNA replication and are dismantled and then restored during replication fork movement while preserving epigenetic information through histone recycling to maintain cell identity [25, 26].

The molecular machinery that replicates DNA in eukaryotic cells (known as replisome) is assembled at origins anew every cell cycle in two steps, origin licensing and origin firing, during which replicative helicases are loaded and activated at origins, respectively [27, 28, 29]. The Minichromosome Maintenance 2‐7 (Mcm2‐7 or MCM) complex constitutes the molecular motor of the eukaryotic helicase and is loaded onto double‐stranded DNA in late M and G1 cell cycle phases by the Origin Recognition Complex (ORC), Cdc6, and Cdt1 [30, 31, 32]. With the help of specialized firing factors, Mcm2‐7 is then converted to the active CMG helicase that serves as a platform for replisome assembly upon S phase entry [33, 34, 35]. During this second initiation step, origin DNA is melted. Finally, the two CMG helicases assembled at each origin move in opposite directions, unwinding the parental DNA duplex and allowing DNA polymerases to synthesize leading and lagging strands during replication elongation [36, 37]. Reconstitution of these events in vitro with purified *S. cerevisiae* proteins fueled many biochemical, cryo‐electron microscopy (cryo‐EM), and single‐molecule studies that revealed replication initiation mechanisms at unprecedented molecular detail [38]. While these efforts have been dominated by working with the *S. cerevisiae* system, recent successes in reconstituting origin licensing with purified human proteins pave the way to ask how replication initiation mechanisms may deviate from the paradigms established in budding yeast [39, 40, 41]. Here, we will discuss our current understanding of the molecular choreography underlying the licensing of origins in higher eukaryotes and highlight differences to yeast. Readers interested in origin firing mechanisms, initiation site regulation and function, and replisome activities are encouraged to refer to recent reviews covering these topics [34, 35, 36, 37, 42].

## 2 Loading of the First MCM Hexamer

**FIGURE 1:**
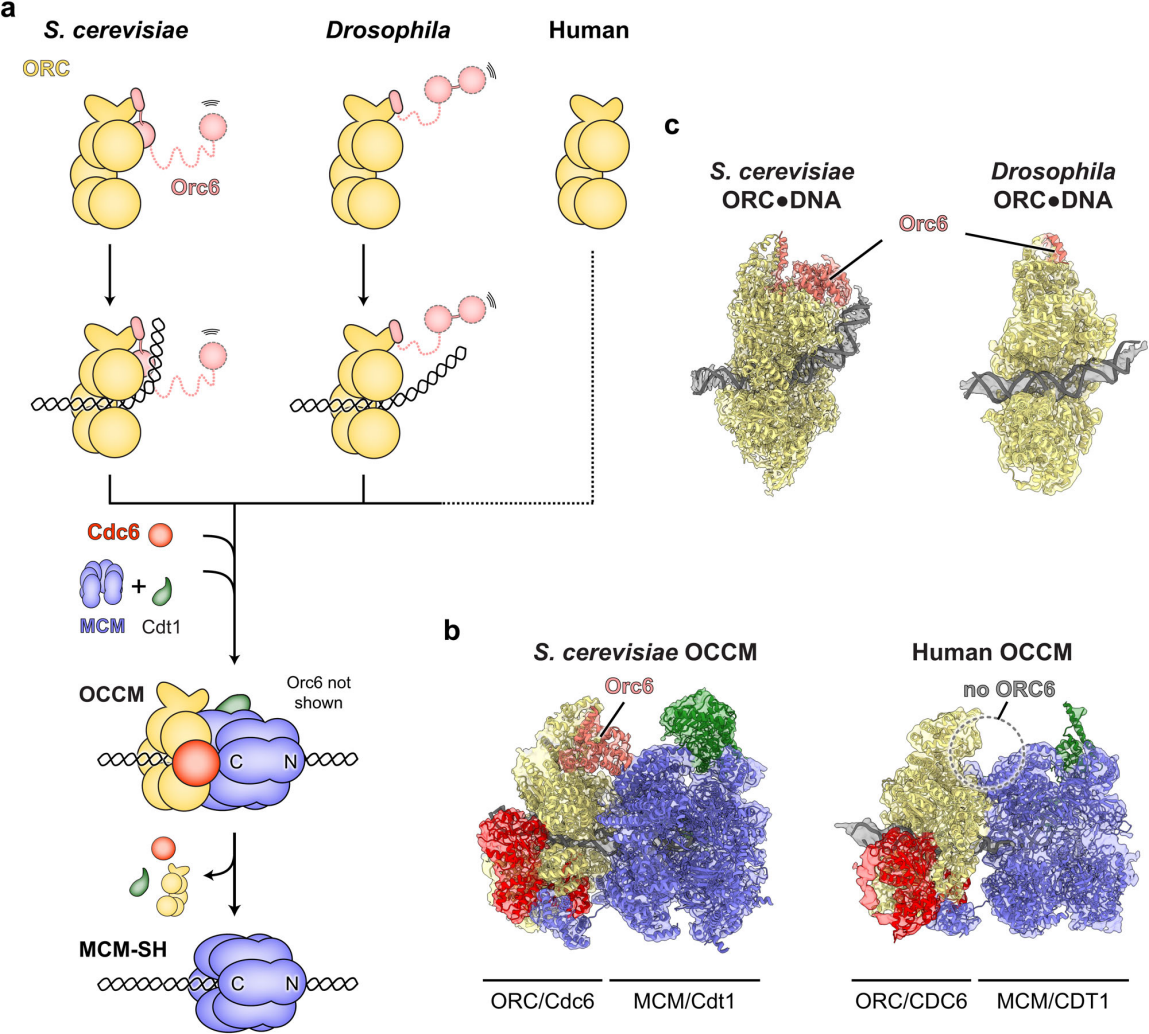
Loading of the first MCM hexamer. (a) Schematic of MCM single hexamer (MCM‐SH) loading. ORC first binds and bends DNA, then recruits Cdc6, Cdt1, and MCM to form an OCCM intermediate, and loads an MCM hexamer onto DNA. Yeast and *Drosophila* ORC form an integral hexameric complex, whereas human ORC assembles as a stable pentameric complex lacking ORC6. (b) Structures of *S. cerevisiae* (EMDB‐8540, PDB 5V8F [52]) and human OCCM (EMDB‐19566, PDB 8RWV [41]) intermediates. (c) Structures of *S. cerevisiae* (EMDB‐6941, PDB 5ZR1 [56]) and *Drosophila* ORC (EMDB‐22362, PDB 7JK5 [61]) bound to DNA, illustrating different extents of DNA bending and distinct contributions of Orc6.

Origin licensing culminates in the loading of two MCM hexamers onto DNA to form double hexamers [43, 44, 45]. Interactions between both MCM rings in the double hexamer are mediated by MCM's N‐terminal domains, with the two MCM hexamers positioned in opposing orientations on DNA [43, 46]. This organization imprints bidirectionality of DNA replication into the MCM double hexamer; each MCM ring will eventually comprise the motor of one of the two CMG helicases that propel replication forks away from the origin during elongation [47, 48]. Both MCM hexamers in the double hexamer are deposited in separate loading events, each choreographed by ORC, Cdc6, and Cdt1 [49, 50] (Figure 1a). ORC and Cdc6 are first recruited to origins and assemble into a hexameric ring that encircles DNA and provides a docking site for an MCM hexamer [51, 52]. The resultant OCCM intermediate also contains Cdt1 and is responsible for loading an MCM single hexamer onto DNA [50, 51]. Cryo‐EM structures of OCCM complexes have revealed similar organizations of both yeast and human intermediates, although some differences exist regarding which MCM subunits contact ORC through their C‐terminal winged‐helix domains [40, 41, 52] (Figure 1b). In the OCCM, human CDT1 engages the MCM ring in a similar manner as does yeast Cdt1, despite an inability of human MCM and CDT1 to co‐associate into a heptameric CDT1‐MCM complex prior to OCCM assembly as seen in yeast [39, 40, 41, 43, 52, 53, 54]. The overall mechanism of loading a single MCM hexamer is likely conserved, with ORC‐CDC6 directing the insertion of DNA into the central MCM pore through an opening in the MCM ring between subunits MCM2 and MCM5 [55].

Unlike in budding yeast, not all MCM loading factors are strictly required for recruiting and loading human MCM hexamers onto DNA in vitro. Omission of human CDC6 or CDT1 still allowed MCM recruitment to DNA, and the formation of OCCM‐like complexes (lacking either CDC6 or CDT1), and MCM double hexamers could form in low‐salt conditions without CDC6 in one of these studies [39, 40, 41]. A cryo‐EM structure of an OCCM‐like complex comprised of human ORC, CDT1, and MCM (OCM) but not CDC6 showed MCM engaging in similar interactions with ORC and DNA as in the canonical OCCM [40]. This structure is consistent with MCM hexamer loading without CDC6, although it could also correspond to an OCCM disassembly intermediate after CDC6 has been released. The most unexpected finding from the in vitro reconstitution of human origin licensing, however, is that ORC6, one of the ORC subunits, is neither required for efficient MCM recruitment nor for MCM loading [39, 40, 41]. In yeast, Orc6 is tightly associated with the other ORC subunits and stabilizes a DNA bend that helps align the DNA duplex with the Mcm2/5 gate for insertion into the MCM hexamer [56, 57, 58] (Figure 1c). This role does not appear to be conserved in metazoan Orc6, suggesting that DNA insertion into metazoan MCM hexamers may be more reliant on the flexibility of a DNA duplex to sample different conformations spontaneously [59, 60, 61] (Figure 1c). Indeed, local DNA flexibility affects the loading efficiency of *Drosophila* MCM in vitro, and the recent reconstitution of human MCM loading provides an opportunity to test this attribute in other metazoan systems as well [61]. Thus, although substantial parallels exist, the requirements for MCM single hexamer loading appear more relaxed in metazoan species compared to yeast, with Orc6 not partaking in this step.

MCM single hexamer loading is coupled to ATP hydrolysis by MCM [62, 63, 64, 65]. All MCM subunits are AAA+ ATPases that form six composite ATPase sites at the subunit interfaces [66, 67]. Biochemical, structural, and single‐molecule analyses of yeast MCM loading indicate that ATP hydrolysis by MCM fuels MCM pore loop movements and translocation on DNA, Cdt1 release, and a relative rotation of MCM's N‐terminal domain (NTD) and AAA+ rings [52, 62, 63, 64, 65, 68]. Collectively, these conformational changes promote full MCM gate closure and OCCM disassembly, resulting in loaded MCM single hexamers. Conspicuously, human MCM single hexamer loading is likewise accompanied by nucleotide hydrolysis at most ATPase sites as seen in recent cryo‐EM structures of MCM single hexamers loaded onto DNA [39, 40]. Yet, a substantial amount of human MCM hexamers can still be loaded when ATP is replaced by the slowly‐hydrolysable analog ATPγS, but double hexamers form only infrequently [39]. While it remains to be established if OCCM maturation into single MCM hexamers proceeds in the absence of ATP hydrolysis in these instances or if ATPγS can be hydrolyzed in the OCCM, these findings nonetheless raise the possibility that ATP hydrolysis by MCM subunits may drive additional transitions during later steps of human origin licensing.

## 3 Loading of the Second MCM Hexamer and Double Hexamer Formation

**FIGURE 2:**
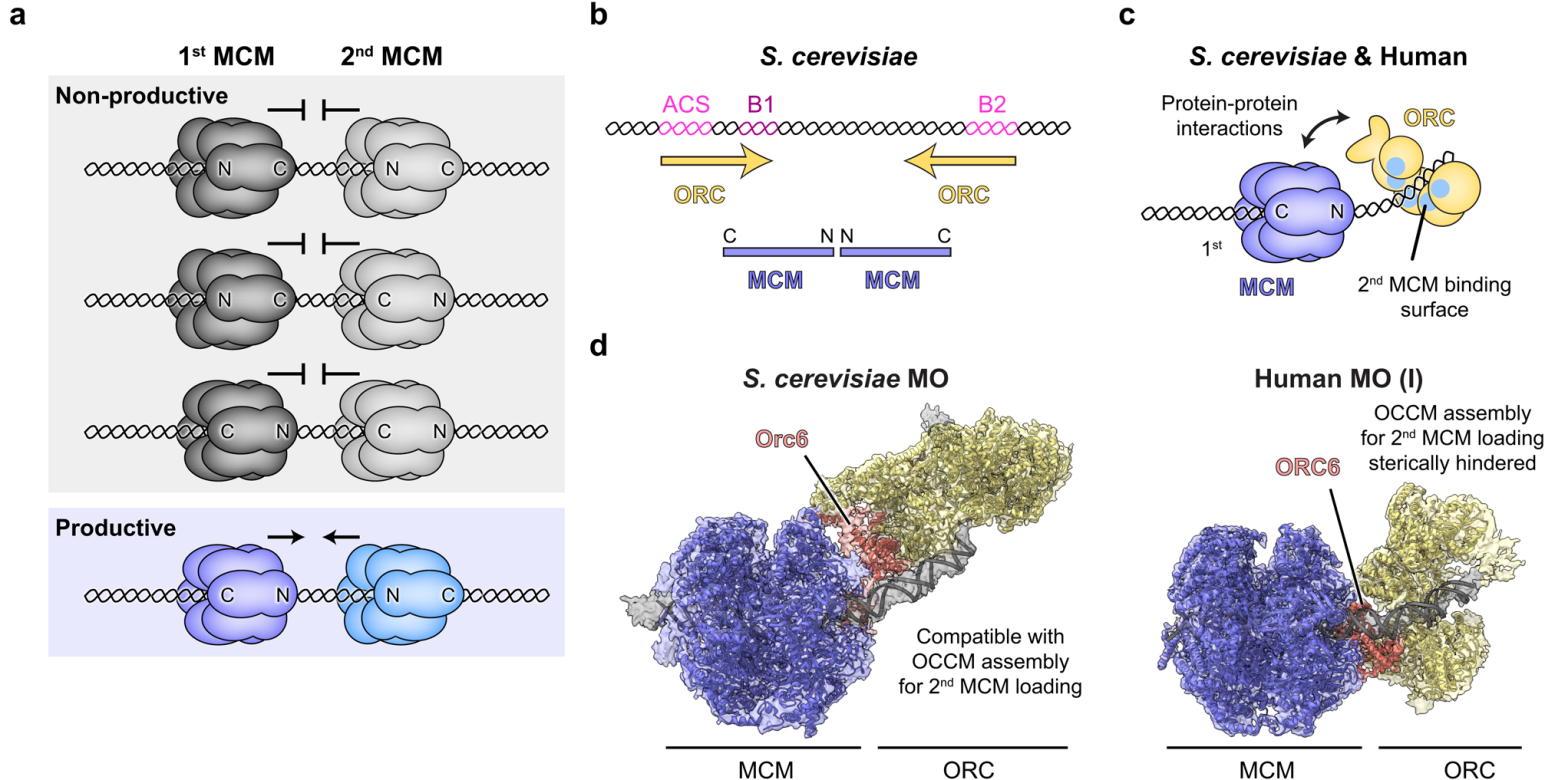
Strategies for orienting the second MCM hexamer during loading. (a) Possible outcomes of independent MCM loading events where the orientation of each hexamer is determined by chance. Only one in four events would result in productive N‐N orientation of loaded MCM hexamers. (b) DNA sequence elements − ACS (ARS consensus sequence), B1, and B2 − at *S. cerevisiae* origins promote directional ORC binding and MCM loading. The arrows and bars indicate positions of ORC and MCM single hexamers, respectively. (c) Protein–protein interactions between ORC and the first loaded MCM hexamer orient ORC on DNA for loading of a second, inverted MCM. (d) Structures of *S. cerevisiae* (EMDB‐4980, PDB 6RQC [65]) and human (EMDB‐19622, PDB 8S0D [40]) MCM–ORC (MO) intermediates.

Recruitment and loading of the second MCM hexamer occur by mechanisms analogous to those of the first and proceed through an OCCM intermediate [49, 50]; however, successful double hexamer formation requires that the second MCM ring is loaded in the opposite orientation to the first. If both MCM hexamers were deposited onto DNA in random orientations, at most 25% of MCM pairs would be compatible with productive head‐to‐head (N‐N) interactions that establish the MCM double hexamer architecture, whereas the other 75% of MCM dimerization attempts (between C‐C, N‐C, and C‐N MCM pairs) would be futile (Figure 2a). Directional deposition of MCM single hexamers onto DNA with their N‐terminal domains facing each other could be achieved either by protein‐DNA interactions or by protein–protein interactions, or a combination thereof, and there is evidence that both strategies are used in eukaryotes to ensure effective origin licensing (Figure 2b–c).

**FIGURE 3:**
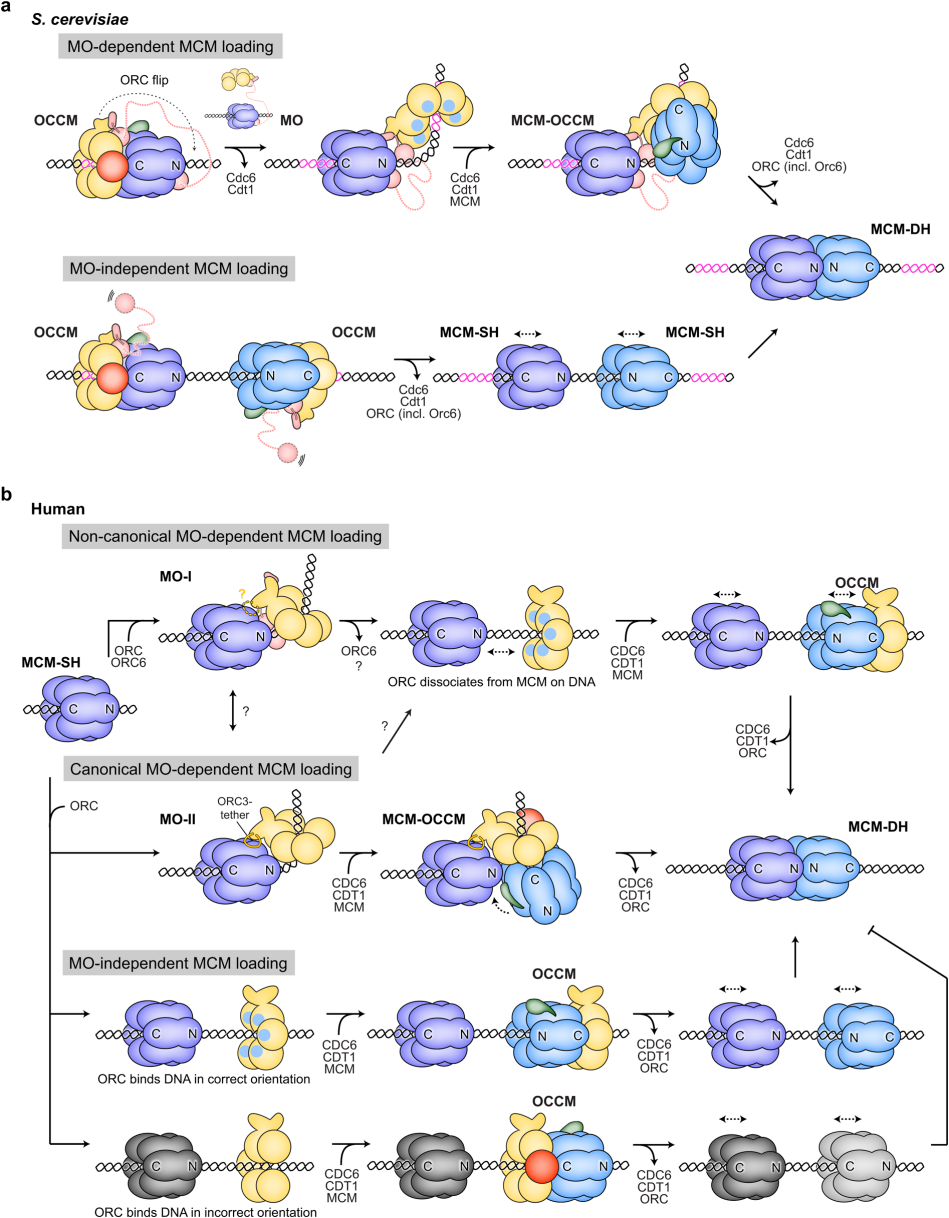
MCM loading in yeast (in a) and human (in b) systems. Mechanisms for MCM double hexamer (MCM‐DH) formation differ with regard to assembly of an MO intermediate and the mode of loading the second MCM hexamer. For yeast, ORC refers to the hexameric Orc1‐6 complex. For humans, ORC denotes the pentameric complex comprised of ORC1‐5. The color scheme is the same as in previous figures.

In budding yeast, replication origins harbor specific DNA sequences that are recognized by ORC and guide directional OCCM assembly and MCM single hexamer loading [56, 69, 70]. Abrogating or altering DNA sequence‐based origin recognition of yeast ORC impedes growth and leads to activation of the intra‐S‐phase checkpoint [71, 72]. While ORC binding to the ARS consensus sequence (ACS) and B1 element mediates loading of the first MCM ring, the B2 element recruits ORC in an inverted orientation to load the second MCM hexamer [65, 73] (Figure 2b). Additionally, direct protein–protein interactions between ORC and the N‐terminal side of the first loaded MCM hexamer, mediated by Orc6, help reinforce the inverted ORC orientation for second MCM recruitment [65] (Figure 2d, left). These contacts are established in the MCM–ORC (MO) intermediate, which then associates with another Cdc6 and Cdt1 to load the second MCM ring (through an MCM–OCCM complex) onto DNA and correctly positions the second MCM for productive dimerization [50, 65] (Figure 3a). The MO is a key licensing intermediate in yeast, and preventing MO assembly impairs MCM loading and cell viability [65, 74, 75, 76, 77]. Indeed, specific inhibition of MO assembly by cyclin‐dependent kinase (CDK)‐mediated phosphorylation of ORC in S‐phase is one strategy yeast cells use to prevent re‐licensing of origins and re‐replication of DNA [76, 78]. Nonetheless, a subset of yeast origins can be efficiently licensed in an MO‐independent manner, indicating these origins solely rely on DNA sequence to drive directional loading of the two MCM rings in the double hexamer [76] (Figure 3a).

Contrary to budding yeast, MCM loading sites in metazoa are not defined by consensus DNA sequence elements, nor are they needed for efficient DNA replication initiation or for accurate regulation [8, 14, 16, 79, 80, 81, 82, 83, 84, 85, 86, 87]. Thus, metazoan origin licensing relies on protein–protein interactions to specify the relative orientation of the two MCM hexamers loaded on DNA. This view is supported by the recent biochemical reconstitution and structural characterization of human origin licensing, which uncovered MO‐like intermediates that can orient human ORC for loading of the second MCM hexamer [39, 40]. As in yeast, assembly of one of the human MO complexes (MO‐I) is mediated by ORC6, but its architecture differs from the yeast MO (Figure 2d, right). Human ORC6 makes distinct contacts with other ORC subunits and MCM, which are sterically incompatible with docking of a second MCM ring onto ORC in MO‐I as in yeast. To support second MCM loading, human ORC and MCM in MO‐I either would need to dissociate and slide apart on DNA prior to loading the second MCM ring, or MO‐I would need to change its conformation (Figure 3b). Indeed, a second human MO intermediate (MO‐II) has been observed in which ORC is tethered to the N‐terminal side of a loaded MCM hexamer through interactions between ORC3 and MCM2, which leaves sufficient room to possibly fit a second MCM hexamer for loading through an MCM–OCCM complex as in yeast [39]. In vitro, both human MOs independently and additively contribute to origin licensing, suggesting they may mediate different MCM loading mechanisms, but crosstalk between these pathways through conversion between the two MO states is likely [39] (Figure 3b). Surprisingly, human MCM double hexamer assembly can still proceed when the MO step is bypassed, albeit less efficiently, as only a subset of loaded MCM single hexamer pairs would be positioned in the correct N to N direction [39]. Thus, there appear to be at least three different mechanisms for second MCM hexamer loading in the human system [39, 40] (Figure 3b); this greater versatility as compared to budding yeast, which likely extends to other metazoan systems as well, may reflect an adaptation to the use of DNA sequence‐independent origins in multicellular eukaryotes.

Completion of origin licensing requires dimerization of the two opposing MCM rings into a double hexamer. In the human but not the yeast complex, DNA is melted at the dimerization interface, although the significance of breaking one base pair for subsequent replication initiation steps is unclear [39, 40, 88, 89, 90]. During double hexamer assembly, extensive interactions are established between the zinc finger domains and N‐terminal insertions and extensions of MCM subunits to interlock both MCM rings and stabilize MCM on DNA, but how exactly this interface forms remains ill‐defined [46]. For example, is additional ATP hydrolysis required for this final licensing step? Do the inter‐subunit contacts between the stacked hexamers need to occur in a defined order? Interestingly, the largest surface area buried in the double hexamers is between Mcm5/3/7 [46]; these subunits can also mediate spontaneous dimerization of open‐ring human MCM hexamers that are not loaded onto DNA and may thus be the driver for dimerization [39]. This notion is consistent with single‐molecule FRET data in yeast showing that Mcm7‐Mcm7 interactions occur well before Cdc6 and Cdt1 release and MCM gate closure during second MCM loading, suggesting that at least a subset of dimerization contacts is formed in the MCM–OCCM intermediate [50]. Nonetheless, MCM loading through MO‐independent pathways in yeast and human systems has shown that dimerization can also occur spontaneously between two loaded MCM hexamers, or in the context of a double OCCM intermediate [39, 40, 76]. Whether inter‐hexamer contacts are formed in a sequential manner and how the correct register is established in such a scenario remain unclear, but it has been suggested that rotational diffusion on DNA may facilitate correct alignment of loaded MCM rings for dimerization [76]. In this regard, it is expected that establishing proper MCM‐DNA contacts in the central MCM pore during single hexamer deposition are key for efficient locking of opposing MCM rings. Future biochemical, biophysical, and structural studies will be needed to resolve these questions and should also address if additional rounds of ATP hydrolysis after MCM single hexamer loading are involved in successful MCM dimerization into double hexamers.

## 4 Inherent Flexibility in Origin Licensing Mechanisms

Multiple lines of research point towards surprising flexibility in origin licensing mechanisms, not only with respect to the mode of determining the correct orientation of the second loaded MCM ring but also regarding the requirements for individual licensing factors and their reuse during MCM loading [39, 40, 76, 91]. This plasticity likely allows adaptation to different origin architectures and local origin contexts. In yeast, the majority of origins are asymmetric with respect to ORC binding affinity (containing one high‐affinity and one low‐affinity ORC site) and are licensed in an MO‐dependent manner, with MO interactions stabilizing ORC on the low‐affinity site [76, 91]. Both DNA binding events can involve the same ORC molecule, which requires ORC to flip over the first loaded MCM hexamer for MO formation, or a second ORC that is recruited into the MO from solution [65, 74]. ORC flipping is expected to be advantageous especially at limiting ORC concentrations when an Orc6‐mediated tether between ORC and MCM increases the probability of ORC binding to the low‐affinity site for second MCM loading [76, 92] (Figure 3a). MO‐independent MCM loading can occur when an origin harbors two high‐affinity ORC sites that each independently load MCM hexamers through a two‐ORC mechanism [76] (Figure 3a). As the distances between ORC sites are variable, several intermediates, including the OCM complex (OCCM after Cdc6 release) and loaded MCM single hexamers, can slide on DNA to accommodate various spacings [76, 91, 93, 94]. Thus, DNA sequences at a given yeast origin play an important role in determining which mechanism is used to license these sites.

Origin licensing in humans displays even greater flexibility than in yeast. At least three distinct mechanisms have been proposed to load human MCM double hexamers: a canonical MO mechanism, a non‐canonical MO mechanism, and an MO‐independent mechanism [39, 40] (Figure 3b). In vitro, ORC6‐mediated MCM loading through the MO‐I intermediate is the most efficient pathway at limiting concentrations of licensing factors; however, MCM loading without ORC6 becomes more productive when protein concentrations are increased [39]. The concentration of each loading factor at an origin likely determines the flux through the various human licensing pathways. At present, local intranuclear concentrations are unknown but may be well above those used in typical in vitro experiments due to the presence of intrinsically disordered regions in ORC, CDC6, and CDT1 that tend to undergo liquid‐liquid phase separation [95, 96]. All human pathways probably use a two‐ORC mechanism as metazoan ORC6 does not appear to contain a sufficiently long linker to mediate an ORC flip as in yeast [39]. Biophysical measurements in cells and visualizing MCM loading directly at intranuclear origins will be critical to determine the relative contributions of the different routes to MCM double hexamers in human cells. Nonetheless, it is worth noting that mutations in several MCM loading factors (including ORC6, CDC6, and CDT1) found in patients with a form of primordial dwarfism, Meier–Gorlin syndrome, cause relatively mild replication defects in cells, consistent with an ability to license origins along multiple pathways [97, 98, 99, 100, 101]. The plasticity of human origin licensing mechanisms stands to increase robustness and resilience of the system, ensuring enough MCM double hexamers are loaded for complete genome replication.

## 5 Origin Licensing Without ORC?

As the initiator, ORC has long been thought to be essential for origin licensing across eukaryotes, a view supported by genetic experiments in yeast, flies, and mammalian cells, as well as biochemical experiments in various model systems [102, 103, 104, 105, 106, 107, 108, 109, 110, 111, 112, 113]. Remarkably, several studies have recently suggested that ORC may be bypassed in certain situations, provoking debates on whether origins can be licensed without ORC [114, 115, 116, 117]. Knockouts of ORC1, ORC2, ORC5, or ORC2/ORC5 in human colon cancer cells yielded viable clones that were able to load MCM onto DNA and replicate their genomes [114, 115]. Origin licensing in these instances is still dependent on CDC6 (and probably also CDT1), and the licensing capacity of cancer cells that lack ORC can support the activation of dormant origins when replication is challenged by genotoxic stresses, consistent with an excess of MCM double hexamers loaded [114, 116]. Although primary cells rely on ORC for MCM loading in the context of the mitotic cell cycle, and potentially cancer cells can license origins with much lower levels of ORC, there is precedent for efficient DNA replication without ORC or with severely reduced ORC levels. *Drosophila* salivary gland cells can undergo endoreduplication, i.e., multiple DNA replication cycles without cell division, in *orc1^−/−^* larvae and accumulate polyploid cells [118]. Similar observations were made in *orc6^−/−^* larvae, but low amounts of maternal ORC persisted and could potentially drive replication [119]. Nonetheless, the concept of origin licensing without ORC was recently extended to polyploid mammalian cells, where mouse trophoblasts and hepatocytes support endoreduplication without ORC [120, 121]. Importantly, the same mutant ORC alleles inhibited genome replication during mitotic proliferation, arguing against substantial residual levels of functional ORC being produced.

If genome replication in cancer cells and polyploid tissues can indeed occur without ORC, what then is the mechanism by which origins are licensed to initiate DNA replication in these instances? It has been proposed that subcomplexes of ORC may retain activity in MCM loading, or that CDC6 could replace specific subunits, such as ORC1, in the initiator complex [114, 122]. However, molecular and structural properties of ORC and ORC‐containing intermediates, as well as multi‐subunit ORC knockout data, make these scenarios unlikely [115]. Moreover, in vitro biochemical experiments have shown that ORC lacking ORC1 is non‐functional for MCM loading even when CDC6 is in large excess [39]. It is conceivable that a yet unidentified MCM loading pathway, possibly using an alternate MCM loader, may be functional in endoreplicating tissues and is selected for in ORC knockout cancer cell lines. Alternatively, replication could initiate in a non‐canonical manner. Of note, some microbial eukaryotes possess an incomplete set of DNA replication proteins and lack a subset of ORC subunits, Cdc6, Cdt1, or all three but retain MCM, consistent with alternative initiation mechanisms, and recombination‐dependent replication initiation has been reported in some archaea [123, 124]. Ultimately, definite proof that DNA replication can initiate without ORC in metazoan systems awaits identification of an alternate MCM loader and/or biochemical reconstitution of ORC‐independent MCM loading.

## 6 Origin Licensing in Chromatin

Chromatin, not free DNA, is the natural substrate for genome duplication and exerts both direct and indirect effects on DNA replication initiation. Undeniably, chromatin context plays a key role in regulating origin licensing, origin selection, and the timing of origin activation [1, 125, 126, 127]. Globally, packaging of DNA into nucleosomes by histones and into higher‐order chromatin structures, combined with the abundance of other DNA‐binding proteins in the nucleus of eukaryotic cells, limits the accessibility to DNA and thereby pose challenges to licensing origins and to replicating DNA in general [25, 128, 129]. For example, the DNA footprint of loaded MCM double hexamers is ∼60 bp, but additional free DNA is needed for assembling loading intermediates (e.g., a minimum of ∼80 bp for an OCCM‐MCM and ∼100 bp for a double OCCM) [39, 40, 46, 52]. Locally, chromatin harbors different epigenetic modifications that can play important roles in recruiting origin licensing factors. Since replication machineries need to be assembled distributively across the entire genome to support complete genome replication, various strategies help cope with different chromatin contexts, such as euchromatin and heterochromatin [130]. The molecular details governing the recruitment mechanisms of initiation factors to chromatinized DNA and the establishment of origin chromatin architectures are beginning to be elucidated.

**FIGURE 4:**
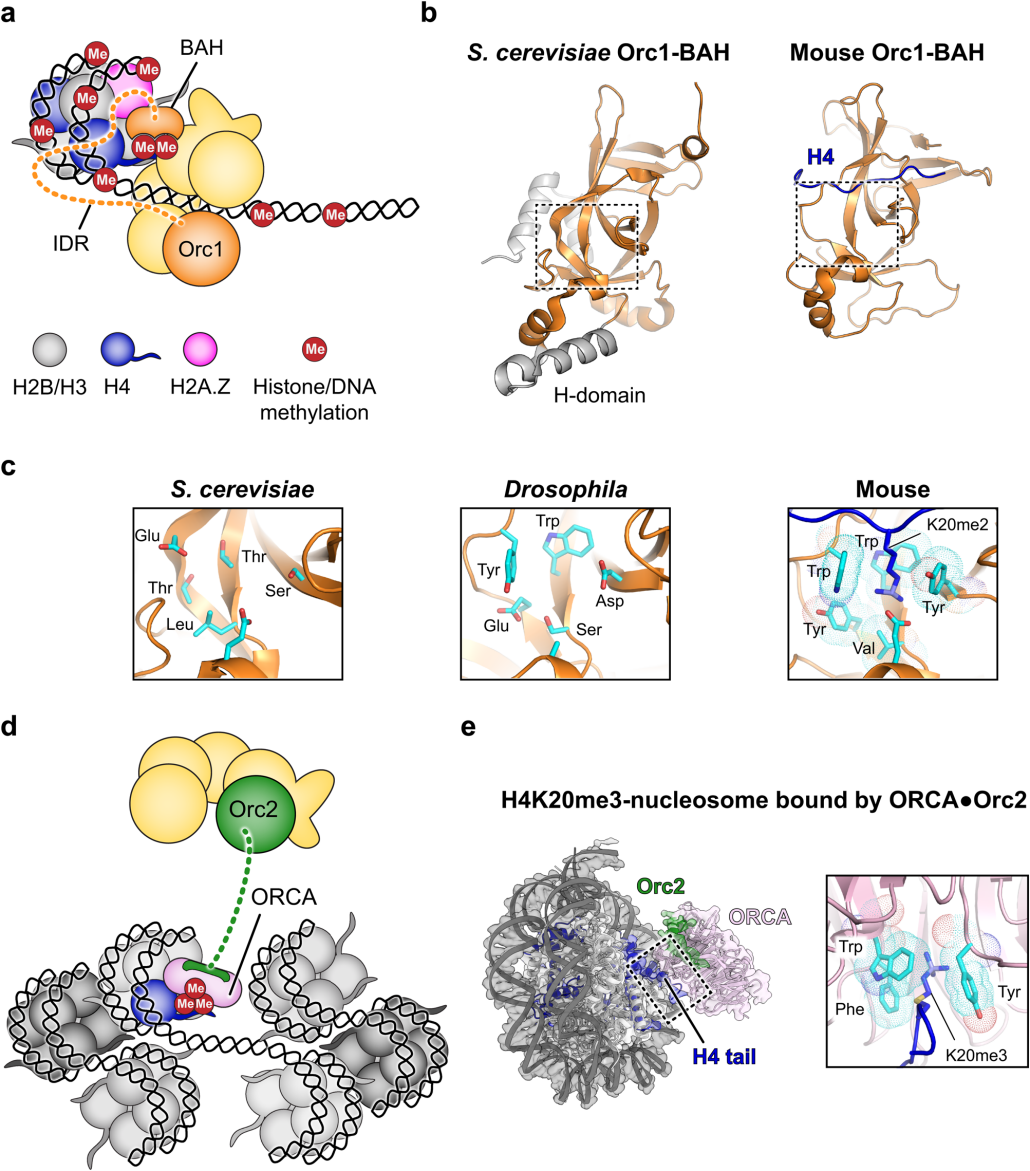
Modes of ORC‐nucleosome interactions. (a) Schematic of a potential ORC‐nucleosome complex. Orc1 binds chromatin through its IDR region and the Orc1‐BAH domain, which recognizes H4 tails (in mammals with H4K20 dimethylation). Epigenetic marks implicated in aiding ORC recruitment in higher eukaryotes are shown. (b) Structure of *S. cerevisiae* (PDB 1M4Z [135]) and mouse (PDB 4DOW [137]) Orc1‐BAH domains. Non‐conserved regions in the yeast structure are colored grey. (c) The aromatic cage (cyan) in the mouse Orc1‐BAH domain that recognizes H4K20me2 is not present in yeast, rationalizing why yeast Orc1‐BAH binds unmethylated but not methylated H4. Zoomed views of regions marked by dashed boxes in **b** are shown. An AlphaFold3 model of *Drosophila* Orc1‐BAH shows only partial preservation of aromatic cage residues [190]. (d) ORCA recruits ORC to chromatin with repressive H4K20me3 marks. (e) Structure of a complex containing an H4K20me3‐nucleosome and ORCA (EMDB‐40522, PDB: 8SIY [163]). Zoomed view of the region in the dashed box shows ORCA's aromatic cage binding H4K20me3.

In both yeast and metazoan cells, interactions with nucleosomes contribute to localizing the initiator ORC to origins, which is mediated by a bromo‐adjacent homology (BAH) domain in ORC's Orc1 subunit [131, 132, 133, 134] (Figure 4a). Despite divergence of the BAH domain in sequence, structure, and specific function between yeast and metazoa, this region directly binds nucleosomes in both cases [135, 136, 137] (Figure 4b). The *S. cerevisiae* BAH domain does not appear to recognize specific histone modifications and collaborates with ARS elements, which are the primary determinants of origin location, to enrich ORC at origins [69, 136, 137, 138]. Some yeast origins are sensitive to BAH deletions, indicating that the BAH domain can modulate origin activity [134]. In mammals, the BAH module in ORC1 specifically recognizes the H4K20me2 modification in nucleosomes [137, 139]. This difference to yeast is rooted in a structural element, an aromatic cage, that is absent in *S. cerevisiae* Orc1‐BAH [137] (Figure 4c). Although mammalian ORC is recruited to H4K20me2, it is unlikely sufficient to determine origin location since 85% of histone H4 bear this modification [140]. Thus, since metazoan origins are not defined by consensus DNA sequences, additional chromatin features or structural DNA elements are likely involved in determining mammalian origin location (Figure 4a). Recently, it has been found that the histone variant H2A.Z is enriched with H4K20me2 at origins, particularly early replicating ones, which has been attributed to H2A.Z‐mediated stimulation of H4 tail modification by the methyltransferase SUV420H1 [141, 142, 143, 144]. Whether the BAH domain or other ORC regions also recognize differences between H2A and H2A.Z that facilitate initiator recruitment to H2A.Z/H4K20me2 nucleosomes remains to be determined. In other metazoa, the situation surrounding Orc1‐BAH and H4K20 methylation appears to be more complex; in *Drosophila*, DNA replication can proceed without H4K20 methylation and Orc1‐BAH deletion does not impede ORC chromatin association, likely due to incomplete conservation of the Orc1‐BAH aromatic cage, while *C. elegans* Orc1 lacks the BAH domain entirely [95, 145, 146] (Figure 4c). Instead, an intrinsically disordered region (IDR) in Orc1 is essential for ORC localization to chromatin in *Drosophila* cells, and related Orc1‐IDRs from other metazoan species can also self‐associate with chromosomes [95, 147]. These observations are consistent with a multifactorial nature of defining metazoan replication origins. Indeed, multiple epigenetic cues have been reported to correlate with replication start sites, many of which mark open chromatin, including H3 and H4 acetylation, H3K4 methylation, DNAse I hypersensitivity, DNA methylation, and G4 quadruplex elements, making them good candidates for regulating origin function [148, 149, 150, 151, 152, 153, 154, 155].

Establishing active replication origins in repressive heterochromatin is more challenging than in open chromatin due to a higher degree of compaction. Heterochromatin also features a different set of epigenetic signals [156, 157]. Consequently, specialized strategies are needed to recruit origin licensing factors for replication initiation. In higher eukaryotes, an ORC‐associated protein, ORCA or LRWD1, helps accomplish this task [130, 158, 159, 160]. ORCA binds both ORC and nucleosomes with heterochromatic marks H3K9me3, H3K27me3, and H4K20me3, thereby recruiting ORC to these chromatin regions [139, 158, 159, 161, 162, 163] (Figure 4d,e). In vitro, ORCA can also inhibit H4K20me3‐nucleosome array compaction, which may help with MCM loading, and reduced levels of ORCA or H4K20me3 interfere with timely heterochromatic origin licensing and activity [130, 163, 164]. Thus, H4 methylation (H4K20me2 and H4K20me3) is emerging as a key player in ensuring timely replication of various chromatin regions in mammalian cells.

Besides ORCA, other proteins can facilitate ORC binding to chromatin in various metazoan species, including HP1, HOAP, MYB/E2F1, HMGA1a, TRF2, and AlF‐C, but if and how they directly contribute to origin licensing remains poorly understood [165, 166, 167, 168, 169, 170, 171, 172, 173, 174]. Likewise, little is known about whether Cdc6, Cdt1, and MCM engage partner proteins (besides ORC) that facilitate their recruitment to origins in chromatin; these loading factors do not contain any known nucleosome binding domains, although metazoan Cdc6 and Cdt1 have long intrinsically disordered regions that could aid in their localization to chromatin [95, 96]. Since ORC is the first core initiation factor to bind an origin and artificial tethering of ORC to a chromatin locus is sufficient for origin formation, the recruitment of ORC and the establishment of a loading‐competent ORC‐DNA complex are likely major determinants for targeting other MCM loading factors to genomic sites [175].

Successful MCM loading in chromatin requires nucleosome‐free regions to accommodate reaction intermediates on DNA [129, 176, 177]. In yeast, origin sequences disfavor nucleosome assembly, facilitating licensing [25, 178, 179]. Additionally, ORC also aids in establishing a specific chromatin organization at yeast origins characterized by regularly spaced origin‐flanking nucleosomes [177, 178, 180]. ORC's ATPase activity, the Orc1‐IDR and BAH domain, as well as chromatin remodelers (i.e., INO80, ISW1a, ISW2, or Chd1) all contribute to phasing these nucleosomes, but the mechanistic details of how ORC and classic chromatin remodelers cooperate to achieve this outcome remains vague [129, 180]. Nonetheless, proper nucleosome phasing is required for efficient in vitro DNA replication and can rationalize the reduced activity of BAH‐dependent yeast origins, which display altered nucleosome positioning upon Orc1‐BAH deletion [134, 177, 180]. Interestingly, yeast ORC appears to be able to destabilize nucleosomes through eviction of H2A/H2B but not H2A.Z/H2B as seen in single‐molecule experiments, an activity also dependent on the BAH domain and ATP hydrolysis by ORC [181]. It is tempting to speculate that both ORC‐mediated nucleosome destabilization and phasing of origin‐flanking nucleosomes act in concert, but additional work is needed to determine if and how these events may be linked, and how they mechanistically enhance chromatin replication.

As in yeast, a specific chromatin organization is expected to be important for origin function in higher eukaryotes as well. This view is supported by observed co‐associations of chromatin remodelers and modifying enzymes with origins and MCM loading factors. For example, SNF2H, an ISWI‐type remodeler, is recruited to some human origins and interacts with CDT1, and the HBO1 histone acetyltransferase associates with multiple licensing factors, including ORC, CDT1, and MCM2 [182, 183, 184, 185, 186]. It is likely that the stimulation of origin licensing is coupled to promoting a more open chromatin organization at origins, but the precise mechanisms remain unknown. Interestingly, G4 structures exclude nucleosomes, promoting the formation of nucleosome‐depleted regions that are more conducive to origin licensing and subsequent initiation steps, possibly explaining their enrichment at replication origins [155, 187]. Whether metazoan ORC can promote phasing of origin‐flanking nucleosomes or destabilize nucleosomes as in yeast is currently unclear. Considering the various origin licensing mechanisms recently uncovered through the in vitro reconstitution of human MCM loading, an intriguing possibility is that chromatin factors might modulate the assembly of specific licensing intermediates and usage of different mechanisms [39, 40]. ORC‐nucleosome interactions, for instance, may orient ORC on DNA to enforce the loading of opposing MCM single hexamers that are poised for dimerization, thereby enhancing the efficiency of the MO‐independent pathway; alternatively, nucleosomes could act as barriers to MCM hexamer sliding on DNA and MCM single hexamer encounters, inhibiting MO‐independent MCM loading [188]. Future work is clearly required to resolve these exciting questions.

## 7 Conclusions and Outlook

Great strides have been made towards understanding the mechanistic underpinnings of eukaryotic DNA replication initiation. The biochemical reconstitution of the complete DNA replication initiation reaction with purified yeast proteins on DNA and chromatin substrates has illuminated important principles of origin licensing and firing that establish bidirectionality of DNA replication and provided a powerful platform for many structural and single‐molecule studies [25, 38, 189]. While our understanding of metazoan origin definition, licensing, and firing has lagged behind, this disparity is rapidly diminishing as exemplified by the recent in vitro reconstitution of human MCM loading by multiple laboratories and the progress on deciphering MCM loading mechanisms [39, 40, 41]. Nonetheless, important challenges lie ahead. Complete biochemical reconstitution of replication initiation (i.e., origin licensing and origin firing) with purified metazoan proteins has not been realized yet and should clearly be a future goal. Extending these studies to different types of chromatin substrates will help elucidate how epigenetic cues contribute to metazoan origin definition and origin activity. Moreover, in situ studies are needed to test the principles derived from in vitro studies in cells. For example, is the flexibility in MCM loading mechanisms seen in cells as well? New developments in in situ single‐molecule biophysics and structural biology will provide exciting opportunities to tackle these problems and further advance our knowledge of this fundamental process in multicellular organisms.

## Author Contributions

Conceptualization: Franziska Bleichert. Writing − original draft: Franziska Bleichert, Victoria Frisbie. Writing − review and editing: Franziska Bleichert, Victoria Frisbie.

## Conflicts of Interest

The authors declare no conflicts of interest.

## Data Availability

Structure data included in this review are openly accessible in the Protein Data Bank at https://www.rcsb.org/ and the Electron Microscopy Data Bank at https://www.ebi.ac.uk/emdb/.
